# LoReTTA, a user-friendly tool for assembling viral genomes from PacBio sequence data

**DOI:** 10.1093/ve/veab042

**Published:** 2021-04-23

**Authors:** Ahmed Al Qaffas, Jenna Nichols, Andrew J Davison, Amine Ourahmane, Laura Hertel, Michael A McVoy, Salvatore Camiolo

**Affiliations:** 1 Department of Pediatrics, Virginia Commonwealth University, Richmond, VA, USA; 2 MRC-University of Glasgow Centre for Virus Research, Glasgow, UK; 3 Department of Pediatrics, School of Medicine, University of California San Francisco, Oakland, CA, USA

**Keywords:** de novo assembly, viral genomics, long read assembler, PacBio

## Abstract

Long-read, single-molecule DNA sequencing technologies have triggered a revolution in genomics by enabling the determination of large, reference-quality genomes in ways that overcome some of the limitations of short-read sequencing. However, the greater length and higher error rate of the reads generated on long-read platforms make the tools used for assembling short reads unsuitable for use in data assembly and motivate the development of new approaches. We present LoReTTA (Long Read Template-Targeted Assembler), a tool designed for performing *de novo* assembly of long reads generated from viral genomes on the PacBio platform. LoReTTA exploits a reference genome to guide the assembly process, an approach that has been successful with short reads. The tool was designed to deal with reads originating from viral genomes, which feature high genetic variability, possible multiple isoforms, and the dominant presence of additional organisms in clinical or environmental samples. LoReTTA was tested on a range of simulated and experimental datasets and outperformed established long-read assemblers in terms of assembly contiguity and accuracy. The software runs under the Linux operating system, is designed for easy adaptation to alternative systems, and features an automatic installation pipeline that takes care of the required dependencies. A command-line version and a user-friendly graphical interface version are available under a GPLv3 license at https://bioinformatics.cvr.ac.uk/software/ with the manual and a test dataset.

## 1. Introduction

DNA sequencing has prompted a period of explosive growth in the genomics of microorganisms. The method developed by Sanger was used to sequence the genome of bacteriophage φX174 (Sanger et al. 1977) and then the genomes of many other organisms but has been superseded by technologies capable of much higher throughput (Slatko et al. 2018). These technologies rely on producing huge numbers of short or long reads from DNA fragments and assembling them using sophisticated software typically tailored to the sequencing platform employed.

Short-read technologies, such as the Roche 454, Ion Torrent and Illumina platforms, generate highly accurate reads of up to several hundred nucleotides (nt), whereas long-read technologies, such as the PacBio and Oxford Nanopore platforms, produce much longer but less accurate reads (Slatko et al. 2018). Long-read data offer the advantage of resolving regions that are repetitive or otherwise difficult to reconstruct (Pollard et al. 2018) and are also not necessarily dependent on polymerase chain reaction (PCR) amplification and its biases (Potapov and Ong 2017). Powerful approaches have been developed to reduce the impact of high error rates, such as PacBio circular consensus sequencing (CCS; Larsen et al. 2014), and several *de novo* assemblers have been designed that can cope with long, error-prone reads (Amarasinghe et al. 2020). Read assembly is typically based on an overlap-layout-consensus approach that produces long contigs or, ideally, a complete genome by recursively joining overlapping reads. In order to minimize the effect of sequencing errors, the longer reads in a dataset are used as references to align the shorter reads, and errors are corrected by calling the consensus from the alignments (Wick and Holt 2019). The corrected long reads are then joined via their overlaps to form a draft assembly that is finally polished by comparison to the original dataset.

**Figure 1.**
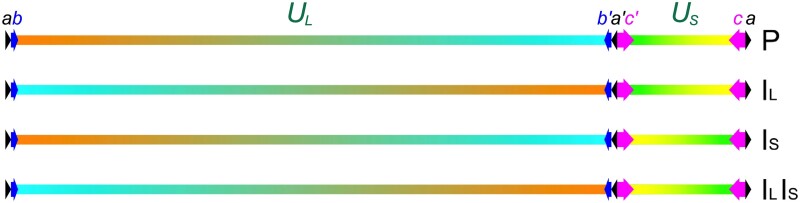
HCMV genome isomers. Arrows represent the locations and orientations of *a* (black), *b* (blue) and *c* (magenta) and their inverted copies, *a′*, *b′* and *c’*. Colour gradients in the unique sequences (U_L_ and U_S_) indicate the relative orientations of these regions in the four isomers, which are designated prototype (P), U_L_ inverted (I_L_), U_S_ inverted (I_S_) and U_L_ and U_S_ inverted (I_L_I_S_). For simplicity, a single copy of the *a*/*a′* sequence is shown at the termini and internally. However, a proportion of genomes has been reported to contain additional directly repeated copies of *a* at the left end and internally or to lack a copy of *a* at the right end.

Our interest is in human cytomegalovirus (HCMV; *Human betaherpesvirus 5*), which is a ubiquitous virus with a large (approximately 236 kbp), linear, double-stranded DNA genome. The complete sequences of many strains of this virus have been determined using short-read technology (Sijmons et al 2015; Suárez et al. 2019), but the size and diversity of the genome have led us to consider the potential of long-read technology as a more efficient approach in some circumstances (e.g. for resolving long repeats or linking variants). This technology has been used to reconstruct the genomes of many organisms (Jain et al. 2018; Li et al. 2018), but its use for viral genomes raises particular challenges because viruses rapidly accumulate genetic variations as a result of their short generation times and, especially in the case of RNA viruses, high mutation rates (Domingo et al. 2012). As a result, heterogeneity due to genuine variation may be difficult to distinguish from that due to error and may compromise genome reconstruction. Moreover, some viral genomes incorporate repeated regions that may also confound genome reconstruction. Thus, the HCMV genome consists of two unique regions (U_L_ and U_S_), each flanked by an inverted repeat (*ab*/*b′a′* and *a′c′*/*ca*), in the overall arrangement *ab*-U_L_-*b′a′c′*-U_S_-*ca* (Stinski 1991) (Fig. 1). The genome is terminally redundant, having a direct repeat (*a*) at its ends that is also present internally as an inverted copy (*a′*), with the added complications that some molecules contain multiple copies of *a*/*a′* at the left end or internally and some lack the copy of *a* at the right terminus (Tamashiro and Spector 1986). Recombination between the inverted repeats in concatemeric genomes during DNA replication followed by cleavage of unit-length genomes from concatemers lead to the co-existence of equimolar amounts of four isoforms differing in the relative orientations of U_L_ and U_S_ (McVoy and Adler 1994) (Fig. 1). These structural features are largely invisible on the scale of short reads, but their representation in long reads can prematurely terminate assembly or introduce artefactual duplications.

Focusing on long-read data generated on the PacBio platform, we found that established assemblers were not successful at reconstructing HCMV genomes, for the reasons outlined above. As a result, we designed LoReTTA (Long Read Template-Targeted Assembler), a new tool for assembling viral genomes from PacBio reads. In contrast to established assemblers, this tool employs a user-provided reference genome to guide assembly, which is an approach that has proved successful with short reads (Lischer and Shimizu 2017). LoReTTA was tested on simulated and experimental datasets for several viruses, and its performance was compared with that of three established *de novo* assemblers and one recently reported reference-guided assembler. Although the software was originally designed to deal with HCMV, it proved equally successful at assembling a range of viral genomes.

## 2. Materials and methods

### 2.1 Read datasets

#### 2.1.1 Simulated datasets

SimLoRD v. 1.0.4 (Stöcker et al. 2016) was used with default parameters to generate simulated PacBio datasets incorporating an appropriate error profile for viral genomes representing a wide range of sizes and nucleotide compositions (Supplementary Table S1). The genomes were those of: HCMV strains Merlin and AD169 (substrain varUC), herpes simplex virus type 1 (HSV-1) strain KOS, hepatitis B virus (HBV) strain ayw, hepatitis C virus (HCV) strain H77 (genotype 1) and strain HC-J6CH (genotype 2), severe acute respiratory syndrome coronavirus 2 (SARS-CoV-2) strain Wuhan-Hu-1 and *Pseudomonas aeruginosa* phage 1 (PaP1). Read datasets were simulated to represent average coverage depths of 100, 500 and 1,000 reads/nt. Dataset identifiers included the abbreviation of the virus name and, if appropriate, the strain name, followed by the average coverage depth in reads/nt (e.g. dataset ${\text{HCMV}}_{\text{Merlin}}^{500}$ represents the dataset derived from HCMV strain Merlin with an average coverage depth of 500 reads/nt). To accommodate the features of the HCMV genome, the simulated reads were generated from an equimolar mixture of reference genomes corresponding to all four isoforms (Fig. 1). This step was also implemented for HSV-1, which has a similar genome structure.

Simulated datasets representing a mixture of the two HCMV strains were generated by mixing reads in various proportions (datasets ${\text{HCMV}}_{\text{mixed}}^{90\text{:}10}$, ${\text{HCMV}}_{\text{mixed}}^{80\text{:}20}$, ${\text{HCMV}}_{\text{mixed}}^{70\text{:}30}$, ${\text{HCMV}}_{\text{mixed}}^{60\text{:}40}$; the ratio indicating Merlin: AD169). Each dataset comprised sufficient strain Merlin reads to yield an average coverage depth of 100 reads/nt.

In addition to datasets originating from a single viral strain or a mixture of two HCMV strains (and nothing else), HCMV datasets containing a high proportion of human reads were generated by diluting simulated HCMV strain Merlin reads (coverage depth 100 reads/nt) at set percentages into a deposited experimental human PacBio dataset (NCBI SRA accession no. SRR11354531; datasets ${\text{HCMV}}_{\text{clinical}}^{2\%}$, ${\text{HCMV}}_{\text{clinical}}^{5\%}$, ${\text{HCMV}}_{\text{clinical}}^{10\%}$ and ${\text{HCMV}}_{\text{clinical}}^{15\%}$). These datasets mimic clinical samples containing a small proportion of target virus in a large background of human material. The same approach was used to generate HCMV datasets containing a small proportion of HCMV reads in a deposited experimental human gut microbiome PacBio dataset (NCBI SRA accession no. ERR4025905; datasets ${\text{HCMV}}_{\text{meta}}^{2\%}$, ${\text{HCMV}}_{\text{meta}}^{5\%}$, ${\text{HCMV}}_{\text{meta}}^{10\%}$ and ${\text{HCMV}}_{\text{meta}}^{15\%}$). These datasets mimic environmental samples containing a small proportion of target virus in a large, complex background of material from other organisms. Owing to the large size of the HCMV genome, a large read dataset (>2 Gb) was produced for the ${\text{HCMV}}_{\text{meta}}^{2\%}$ dataset in order to achieve the required coverage depth. As a result of the high error rate of PacBio sequencing technology, this depth (100 reads/nt) proved generally to be the minimum required by LoReTTA to generate a reliable assembly.

#### 2.1.2 Experimental datasets

LoReTTA was also tested using deposited experimental PacBio datasets for HSV-1, HBV and PaP1 (Supplementary Table S2), in addition to an experimental dataset generated in this study for HCMV strain Ig-KG-H2. This strain had been isolated from urine on human fibroblasts cells, passaged twenty-two times in fibroblasts, cloned by limiting dilution and expanded into a virus stock, all in the presence of hyperimmune globulin (HIG) to neutralize cell-free virus released from infected cells (Ourahmane et al. 2019). The sequence of this virus had been determined from short-read data generated principally on the Illumina platform (Al Qaffas et al. 2020) (GenBank accession no. MN274568.2). The virus was passaged fourteen times more in the absence of HIG, and at the final passage, designated Ig-KG-H2p14s, extracellular virions were pelleted from the cell culture supernatant by ultracentrifugation. DNA was isolated by phenol/chloroform extraction and ethanol precipitation as described previously (Ourahmane et al. 2019) and subjected to PacBio sequencing. HiFi SMRTbell library construction and sequencing were performed using Sequel II System 2.0 with P2/C2 (polymerase 2.0 and chemistry 2.0) chemistry at the Genomics Core at Virginia Commonwealth University according to the manufacturer’s instructions. DNA quality was evaluated using the Femto Pulse System (Agilent), and DNA concentration was quantified using a Qubit fluorometer (Thermo Fisher Scientific). The DNA samples were concentrated and purified by adding 0.45 volumes of AMPure PB magnetic beads (PacBio). Sequencing libraries were prepared using a SMRTbell™ Express Template Prep Kit 2.0. The libraries were combined and size-selected electrophoretically using BluePippin Systems (SAGE Science) with the 0.75% DF Marker S1 high-pass 6-10kb vs3 cassette. After pooling the appropriate size fractions, the libraries were further cleaned and concentrated by adding 0.55 volumes of AMPure PB magnetic beads. The concentration of the library pool was measured using a Qubit 1X dsDNA HS assay kit, and the final size distribution was confirmed on the Femto Pulse System. A total of 41,690 polymerase reads were generated that included 1,118,396 subreads with a mean read length of 1,300 nt. For analysis, the high error rate of long reads was mitigated by implementing the CCS protocol (https://www.pacb.com/wp-content/uploads/SMRT-Tools-Reference-Guide-v8.0.pdf). Briefly, an unaligned bam file containing the raw reads was processed using pbindex, generating an XML-formatted index. This index was used to generate high quality (HiFi) reads in a compressed fastq format by implementing CCS with the arguments ‘–minPredictedAccuracy .99’, ‘–richQVs’ and ‘–polish’. This protocol reduces the error rate of PacBio reads to less than one per cent (Rhoads and Au 2015) and resulted in a final dataset of 29,340 reads with an average length of 1,627 nt.

To obtain additional sequence data with a low error rate, an aliquot of the same DNA sample was subjected to short-read sequencing at the MRC-University of Glasgow Centre for Virus Research. Briefly, 100 ng DNA was sheared using a Covaris S220 sonicator to an approximate size of 450 nt and then processed into a sequencing library by carrying out seven cycles of PCR with New England Biolabs indexed primers and a Kapa LTP library preparation kit. The library was sequenced in an v. 2 300-cycle cartridge on an Illumina MiSeq, generating 6,693,058 paired-end 150 nt reads.

The PacBio and Illumina datasets were deposited in the European Nucleotide Archive database under accession numbers ERR5052619 and ERR5052486, respectively.

### 2.2 Benchmarking

The simulated and experimental PacBio datasets were assembled using LoReTTA and the appropriate reference genome (Supplementary Table S1). The quality of each assembly was compared with that of the assemblies produced by three established long-read assemblers: Canu v. 2.0 (Koren et al. 2017), Flye v. 2.8 (Kolmogorov et al. 2019) and Raven v. 1.1 (Vaser and Mile 2020). These tools were used with default parameters, except that (1) the flags ‘–pacbio-corrected’ and ‘–pacbio-corr’ were used to input the reads to Canu and Flye, respectively, (2) the expected genome size was passed to these two assemblers using the flags ‘genomeSize=’ and ‘–genome-size’, respectively, and (3) the flag ‘useGrid=False’ was used to disable grid computing in Canu. The recently reported reference-based long read assembler Rebaler 0.2.0 (Wick et al. 2019) was also tested by running it with default parameters.

Each of the assemblers had the potential to produce a single assembly representing the viral genome from which the reads had been generated. However, several contigs corresponding to fragments of the genome were typically produced. Therefore, the assemblies were compared in terms of the total number of contigs and the N50 value, the latter of which is a measure of assembly contiguity (Earl et al. 2011). Since these parameters indicate completeness but not accuracy, the assemblies were also compared using QUAST v. 5.0.2 (Gurevich et al. 2013). This program reports the level of genome completeness (using either the genome from which the simulated reads had been generated or the deposited genome for the experimental dataset; Supplementary Tables S1 and S2), the number of mismatches and indels per 100 kb, and the number of misassemblies defined as positions in the contigs where the left flanking sequence aligns more than 1 kb distant from the right flanking sequence in the reference genome, or where the flanking sequences overlap by >1 kb or align on different strands. Moreover, the number of supporting reads was computed for the experimental datasets when differences were observed between the LoReTTA-assembled sequence and the corresponding deposited sequence. To achieve this, a forty-one-nucleotide sequence containing the variant nucleotide at the central position was extracted from the LoReTTA assembly and the deposited genome, and the number of occurrences within the reads of each sequence (including its reverse complement) was counted.

## 3. Software implementation

### 3.1 Reference genome subsampling

**Figure 2.**
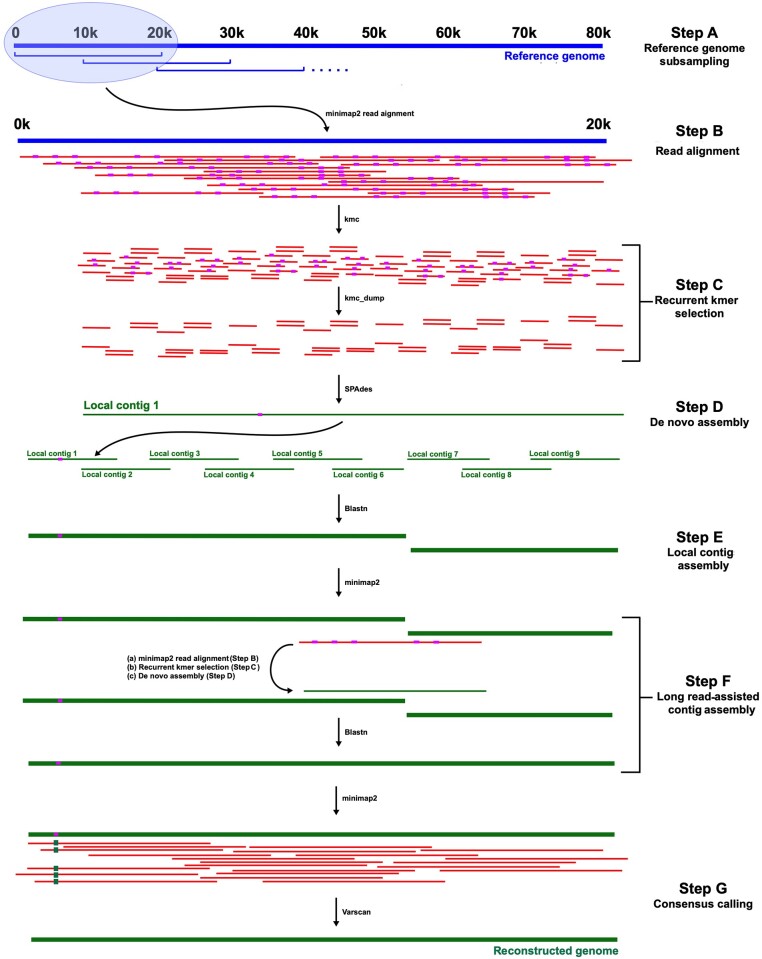
LoReTTA pipeline. Step A: the reference genome (blue; scale in kbp (k)) is subsampled in sliding windows. Step B: reads (red) are aligned to each window (purple segments represent sequencing errors). Step C: all kmers are extracted from each read and the most recurrent are selected. Step D: the selected kmers are used to perform a local contig assembly. Step E: adjacent local contigs are joined by exploiting their overlaps. Step F: gaps due to non-overlapping local contigs are closed using long reads. Step G: the long-read dataset is aligned to the reconstructed genome, and substitutions and indels in more than fifty per cent of reads are emended (green segments represent corrected sequence errors).

A reference genome is used to guide the LoReTTA assembly process (Fig. 2). The genome is subsampled into windows of 20,000 nt that overlap by 10,000 nt (i.e. the first window is 1–20,000 nt, the second is 10,000–30,000 nt, and so on until the entire genome is represented; Fig. 2, step A). For each window, reads are mapped using minimap2 v. 2.17 (Li 2018) (Fig. 2, step B), and those overlapping for more than seventy per cent of their length are extracted from the initial dataset and assembled *de novo* as described in Section 3.2. These window sizes and overlap percentages represent the default parameters and, although adjustable by the user, proved to be suitable for all the datasets tested (even those featuring genomes smaller than the window size, for which step A was not performed). Nonetheless, a smaller overlap percentage can be set when the reference genome diverges significantly from the target genome.

### 3.2 Local *de Novo* assembly

For each window, all possible kmers of sixty-one nucleotide or their complements are extracted from the mapped reads using KMC v. 3.12 (Kokot et al. 2017) (Fig. 2, step C), and those represented more than fifty reads are assembled *de novo* using SPAdes v. 3.14 (Bankevich et al. 2012) (Fig. 2, step D) to produce a sequence (the local contig) representing the window. The rationale is that well-represented kmers can be considered as equivalent to accurate short reads. Indeed, the more frequent a kmer is, the less probable that it contains an error. Finally, if the local contig length is less than eighty per cent of the window size, the kmer count threshold is decreased stepwise from 50 to 2, and then the kmer size of sixty-one nucleotide is decreased stepwise to thirty-one nucleotide, with the count threshold being returned to fifty reads at every kmer size change. This process is terminated as soon as the local contig has adequate length (e.g. 70% of the window size).

### 3.3 Genome reconstruction

Adjacent local contigs are joined via their overlaps, which are identified by aligning them using Blastn v. 2.10 (Altschul et al. 1990) (Fig. 2, step E). Failure to join one or more local contigs will result in gaps, preventing genome reconstruction. In this situation, the original reads are mapped to the two local contigs flanking the gap, and the read aligning to both contigs and featuring the most extended overlaps is selected as a candidate to complete the join (Fig. 2, step F). This read is thus itself used as a reference to perform local *de novo* assembly, and the resulting assembled sequence is used to complete the join via its overlaps with the two flanking local contigs.

### 3.4 Consensus calling

Despite the steps taken to ensure accuracy in Section 3.3, the high error rate of long reads may result in incorporation of erroneous substitutions or small indels. To address this, the dataset is aligned to the reconstructed genome using minimap2 (with the flag ‘-a -x map-pb’) to generate a sam alignment file, which is converted to bam format, sorted and indexed using samtools v. 1.3.1 (Li et al. 2009) (Fig. 2, step G). A pileup file summarizing the base calls at each position in the reconstructed genome is then generated using samtools. Substitutions and indels in this file are called using Varscan v. 2.4.4 (Koboldt et al. 2012), and variants present in more than fifty per cent of the relevant reads are used to emend the reconstructed genome. Homopolymeric regions are challenging to sequence, and reads containing them typically exhibit several length variants. Regardless of whether such variation results from genuine variability or sequencing errors, tests indicated that it is the major contributor to the detection of indels by Varscan. In order to provide the best estimate of homopolymer length, LoReTTA validates all identified indels by counting the number of reads supporting each variant and using the most frequent to emend the reconstructed genome.

## 4. Software evaluation on simulated datasets

### 4.1 Effects of genome size

LoReTTA was tested on simulated long-read datasets generated from viral genomes of known sequence ranging in size from 3,182 to 235,646 nt (Supplementary Table S1), as described in Section 2.1.1. Its performance was compared with that of the assemblers Canu, Flye, Raven and Rebaler. LoReTTA and Rebaler produced a sequence for the smallest genomes (Table 1), HBV (3,182 nt) and HCV (9,646 nt), whereas the other assemblers concluded the elaboration with no output (for Raven) or error messages (‘Failed with exit code 1 (rc = 256)’ for Canu and ‘Error: no disjointigs were assembled’ for Flye).

**Table 1.** Assembly statistics of simulated long-read datasets for four assemblers.

Dataset	N50 (nt)					Contigs (no.)					Ambiguous (‘N’) nucleotides (no.)					Assembled nucleotides (no.)				
	L	R	C	F	Re	L	R	C	F	Re	L	R	C	F	Re	L	R	C	F	Re
${\text{HBV}}_{\text{ayw}}^{100}$	3,162	—	—	—	3,182	1	—	—	—	1	0	—	—	—	0	3,162	—	—	—	3,182
${\text{HBV}}_{\text{ayw}}^{500}$	3,175	—	—	—	3,182	1	—	—	—	1	0	—	—	—	0	3,175	—	—	—	3,182
${\text{HBV}}_{\text{ayw}}^{1000}$	3,175	—	—	—	3,182	1	—	—	—	1	0	—	—	—	0	3,175	—	—	—	3,182
${\text{HCV}}_{\text{gen}1}^{100}$	9,019	—	—	—	9,570	1	—	—	—	1	0	—	—	—	0	9,019	—	—	—	9,570
${\text{HCV}}_{\text{gen}1}^{500}$	9,283	—	—	—	9,550	1	—	—	—	1	0	—	—	—	0	9,283	—	—	—	9,550
${\text{HCV}}_{\text{gen}1}^{1000}$	9,361	—	—	—	9,477	1	—	—	—	1	0	—	—	—	0	9,361	—	—	—	9,477
SARS-CoV-$2_{\text{Wuhan}}^{100}$	28,470	28,927	24,058	29,183	29,500	1	1	1	1	1	0	1	0	0	0	28,470	28,927	24,058	29,183	29,500
SARS-CoV-$2_{\text{Wuhan}}^{500}$	28,558	29,016	18,218	29,004	29,419	1	1	1	1	1	0	1	0	0	0	28,558	29,016	18,218	29,004	29,419
SARS-CoV-$2_{\text{Wuhan}}^{1000}$	28,811	29,054	25,772	28,211	29,519	1	1	1	1	1	0	0	0	0	0	28,811	29,054	25,772	28,211	29,519
${\text{PP}}_{\text{PaP}1}^{100}$	89,878	90,320	61,255	90,490	91,729	1	1	2	1	1	0	1	0	0	0	89,878	90,320	68,019	90,490	91,729
${\text{PP}}_{\text{PaP}1}^{500}$	90,507	91,164	41,996	91,298	91,475	1	1	3	1	1	0	1	0	0	0	90,507	91,164	65,526	91,298	91,475
${\text{PP}}_{\text{PaP}1}^{1000}$	90,726	91,005	11,569	91,126	91,440	1	1	2	1	1	0	1	0	0	0	90,726	91,005	16,853	91,126	91,440
HSV-$1_{\text{KOS}}^{100}$	149,635	129,262	31,652	107,923	152,014	1	2	4	2	1	196	2	0	0	0	149,668	162,428	134,065	151,615	152,014
HSV-$1_{\text{KOS}}^{500}$	150,121	151,163	12,973	138,690	152,332	1	1	5	2	1	169	1	0	0	0	150,116	151,163	66,536	151,614	152,332
HSV-$1_{\text{KOS}}^{1000}$	150,049	136,648	19,618	138,690	152,143	1	2	7	2	1	154	2	0	0	0	150,042	176,004	101,745	151,615	152,143
${\text{HCMV}}_{\text{Merlin}}^{100}$	233,519	206,327	29,649	199,577	235,651	1	2	8	2	1	0	2	0	0	0	233,518	250,544	199,855	235,062	235,651
${\text{HCMV}}_{\text{Merlin}}^{500}$	235,355	235,025	12,645	270,553	235,656	1	1	5	1	1	0	1	0	0	0	235,354	235,025	55,398	270,553	235,656
${\text{HCMV}}_{\text{Merlin}}^{1000}$	234,848	254,672	11,475	270,551	235,651	1	1	7	1	1	0	0	0	0	0	234,848	254,672	78,734	270,551	235,651
${\text{HCMV}}_{\text{mixed}}^{90\text{:}10}$	233,677	264,101	29,407	235,068	235,651	1	1	8	1	1	0	1	0	0	0	233,675	264,101	188,267	235,068	235,651
${\text{HCMV}}_{\text{mixed}}^{80\text{:}20}$	233,675	201,971	29,460	196,437	235,668	1	2	11	3	1	0	2	0	0	0	233,671	254,556	228,380	236,989	235,668
${\text{HCMV}}_{\text{mixed}}^{70\text{:}30}$	234,596	251,519	19,633	231,802	235,454	1	1	15	2	1	1,052	1	0	0	0	234,613	251,519	262,827	236,111	235,454
${\text{HCMV}}_{\text{mixed}}^{60\text{:}40}$	232,884	218,646	16,414	231,982	235,650	1	3	22	2	1	3,407	3	0	0	0	234,750	299,671	325,122	238,019	235,650
${\text{HCMV}}_{\text{clinical}}^{2\%}$	235,646	251,335	—	—	235,650	1	1	—	—	1	0	1	0	0	0	233,518	251,335	—	—	235,650
${\text{HCMV}}_{\text{clinical}}^{5\%}$	233,519	252,791	—	—	235,650	1	1	—	—	1	0	1	0	0	0	233,518	252,791	—	—	235,650
${\text{HCMV}}_{\text{clinical}}^{10\%}$	233,519	251,362	—	—	235,650	1	1	—	—	1	0	1	0	0	0	233,518	251,362	—	—	235,650
${\text{HCMV}}_{\text{clinical}}^{15\%}$	233,519	251,350	—	—	235,650	1	1	—	—	1	0	1	0	0	0	233,518	251,350	—	—	235,650
${\text{HCMV}}_{\text{meta}}^{2\%}$	233,519	202,242	—	—	235,650	1	2	—	—	1	0	1	0	0	0	233,518	262,935	—	—	235,650
${\text{HCMV}}_{\text{meta}}^{5\%}$	233,519	202,242	—	—	235,650	1	2	—	—	1	0	1	0	0	0	233,518	262,935	—	—	235,650
${\text{HCMV}}_{\text{meta}}^{10\%}$	233,519	257,666	—	—	235,650	1	1	—	—	1	0	2	0	0	0	233,518	257,666	—	—	235,650
${\text{HCMV}}_{\text{meta}}^{15\%}$	233,519	257,666	—	—	235,651	1	1	—	—	1	0	2	0	0	0	233,518	257,666	—	—	235,651

–, no assembly was produced when computation was complete (HBV and HCV), or assembly had not finished after an impractical length of time (more than seven days), at which point computation was terminated (HCMV_clinical_ and HCMV_meta_).

L, LoReTTA; R, Raven; C, Canu; F, Flye; Re, Rebaler.

All five tools produced an assembly from the datasets generated from single strains of the larger genomes, although the two reference-guided assemblers often outperformed the other tools in terms of assembly contiguity (i.e. lowest number of contigs and highest N50 values). LoReTTA consistently produced a single contig representing a proportion of the target genome ranging from 93.5 per cent (${\text{HCV}}_{\text{gen}1}^{100}$) to 99.8 per cent (${\text{HBV}}_{\text{ayw}}^{500}$, ${\text{HBV}}_{\text{ayw}}^{1000}$ and ${\text{HCMV}}_{\text{Merlin}}^{500}$). The tool also proved to be very accurate at producing contigs that, in general, did not differ from the target genome in terms of substitutions and indels (Table 2). Several indels were present in the contigs generated from the HSV-1_KOS_ datasets, but the percentage of covered reference genome remained between 98.6 per cent and 98.8 per cent. The proportion of indels was, in general, the lowest among the assemblers except Rebaler and Flye, with the latter generating a more fragmented assembly and a higher number of misassemblies due to the reads representing more than one isoform, as described in Section 4.2. In general, LoReTTA and Rebaler performed very similarly on the simulated datasets (Table 1), with LoReTTA being the more conservative and assembling a smaller number of bases but with fewer errors, especially in terms of indels.

**Table 2.** Comparative statistics for the simulated long-read datasets as determined by the software QUAST using the genomes from which the reads were generated as references.

Dataset	Genome coverage (%)					Mismatches per 100 kb (no.)					Indels per 100 kb (no.)					Misassemblies (no.)				
	L	R	C	F	Re	L	R	C	F	Re	L	R	C	F	Re	L	R	C	F	Re
${\text{HBV}}_{\text{ayw}}^{100}$	99.4	—	—	—	100.0	0.0	—	—	—	0.0	0.0	—	—	—	0.0	0	—	—	—	0
${\text{HBV}}_{\text{ayw}}^{500}$	99.8	—	—	—	100.0	0.0	—	—	—	0.0	0.0	—	—	—	0.0	0	—	—	—	0
${\text{HBV}}_{\text{ayw}}^{1000}$	99.8	—	—	—	100.0	0.0	—	—	—	0.0	0.0	—	—	—	0.0	0	—	—	—	0
${\text{HCV}}_{\text{gen}1}^{100}$	93.5	—	—	—	100.0	0.0	—	—	—	0.0	0.0	—	—	—	10.4	0	—	—	—	0
${\text{HCV}}_{\text{gen}1}^{500}$	96.2	—	—	—	100.0	0.0	—	—	—	0.0	0.0	—	—	—	51.8	0	—	—	—	0
${\text{HCV}}_{\text{gen}1}^{1000}$	97.0	—	—	—	98.2	0.0	—	—	—	0.0	0.0	—	—	—	42.2	0	—	—	—	0
SARS-CoV-$2_{\text{Wuhan}}^{100}$	95.2	96.5	80.0	97.6	98.2	0.0	0.0	25.1	0.0	0.0	0.0	3.5	861.0	0.0	10.2	0	0	0	0	0
SARS-CoV-$2_{\text{Wuhan}}^{500}$	95.5	97.0	60.8	97.0	98.3	0.0	0.0	0.0	0.0	10.2	0.0	0.0	176.0	0.0	20.4	0	0	0	0	0
SARS-CoV-$2_{\text{Wuhan}}^{1000}$	96.3	97.2	86.2	94.3	97.6	0.0	0.0	0.0	0.0	0.0	0.0	6.9	42.7	0.0	3.4	0	0	0	0	0
${\text{PP}}_{\text{PaP}1}^{100}$	98.0	98.5	74.0	98.7	100.0	0.0	0.0	16.2	0.0	1.1	0.0	0.0	374.4	2.2	15.2	0	0	0	0	0
${\text{PP}}_{\text{PaP}1}^{500}$	98.7	99.4	67.5	99.5	99.5	0.0	0.0	19.4	0.0	0.0	0.0	1.1	443.9	0.0	4.4	0	0	0	0	0
${\text{PP}}_{\text{PaP}1}^{1000}$	98.9	99.2	18.3	99.4	99.5	0.0	0.0	29.9	0.0	0.0	0.0	1.1	698.4	0.0	8.8	0	0	0	0	0
HSV-$1_{\text{KOS}}^{100}$	98.6	100.0	87.5	94.0	100.0	0.7	30.9	21.8	0.0	0.7	4.7	157.2	642.8	0.0	2.0	0	2	1	1	0
HSV-$1_{\text{KOS}}^{500}$	98.8	99.5	39.9	95.6	100.0	0.0	0.0	13.2	0.0	0.7	6.0	2.6	385.7	0.0	5.0	0	0	0	1	0
HSV-$1_{\text{KOS}}^{1000}$	98.8	99.8	56.9	95.6	100.0	0.0	5.3	34.7	0.0	0.0	6.0	84.3	723.0	0.0	2.6	0	3	1	1	0
${\text{HCMV}}_{\text{Merlin}}^{100}$	99.1	99.8	83.4	98.9	100.0	0.0	4.3	23.4	0.0	0.0	0.4	44.2	580.3	0.0	0.8	0	0	1	0	0
${\text{HCMV}}_{\text{Merlin}}^{500}$	99.8	98.9	21.0	99.8	100.0	0.0	0.0	28.3	0.0	0.0	0.0	0.9	432.6	0.0	1.3	0	2	0	1	0
${\text{HCMV}}_{\text{Merlin}}^{1000}$	99.7	98.9	29.8	98.9	100.0	0.0	0.4	44.1	0.0	0.0	0.0	10.3	988.2	0.0	2.1	0	2	0	1	0
${\text{HCMV}}_{\text{mixed}}^{90\text{:}10}$	99.2	100.0	77.8	99.8	100.0	0.0	98.0	34.9	0.0	0.0	0.4	135.8	716.3	0.0	3.8	0	3	1	0	0
${\text{HCMV}}_{\text{mixed}}^{80\text{:}20}$	99.2	99.9	87.9	98.9	100.0	0.0	29.3	293.2	0.9	0.0	1.3	74.3	742.4	0.4	1.2	0	1	1	2	0
${\text{HCMV}}_{\text{mixed}}^{70\text{:}30}$	99.0	100.0	84.5	99.4	99.5	24.0	46.3	889.9	9.4	3.4	10.3	62.0	950.2	8.1	7.2	0	2	1	1	0
${\text{HCMV}}_{\text{mixed}}^{60\text{:}40}$	98.0	99.8	92.1	98.1	100.0	325.1	699.0	1321.2	883.4	153.1	29.9	156.9	1267.3	109.8	57.6	0	3	2	1	1
${\text{HCMV}}_{\text{clinical}}^{2\%}$	99.1	100.0	—	—	100.0	0.0	0.4	—	—	0.0	0.4	22.5	—	—	1.3	0	1	—	—	0
${\text{HCMV}}_{\text{clinical}}^{5\%}$	99.1	100.0	—	—	100.0	0.0	10.2	—	—	0.0	0.4	76.8	—	—	1.3	0	2	—	—	0
${\text{HCMV}}_{\text{clinical}}^{10\%}$	99.1	100.0	—	—	100.0	0.0	0.9	—	—	0.0	0.4	24.6	—	—	1.3	0	1	—	—	0
${\text{HCMV}}_{\text{clinical}}^{15\%}$	99.1	100.0	—	—	100.0	0.0	0.4	—	—	0.0	0.4	20.4	—	—	1.3	0	1	—	—	0
${\text{HCMV}}_{\text{meta}}^{2\%}$	99.1	99.8	—	—	100.0	0.0	3.0	—	—	0.0	0.4	44.7	—	—	1.3	0	1	—	—	0
${\text{HCMV}}_{\text{meta}}^{5\%}$	99.1	99.8	—	—	100.0	0.0	3.0	—	—	0.0	0.4	44.7	—	—	1.3	0	1	—	—	0
${\text{HCMV}}_{\text{meta}}^{10\%}$	99.1	100.0	—	—	100.0	0.0	3.8	—	—	0.0	0.4	44.6	—	—	1.3	0	1	—	—	0
${\text{HCMV}}_{\text{meta}}^{15\%}$	99.1	100.0	—	—	100.0	0.0	3.8	—	—	0.0	0.4	44.6	—	—	1.3	0	1	—	—	0

–, no assembly was produced when computation was complete (HBV and HCV), or assembly had not finished after an impractical length of time (more than seven days), at which point computation was terminated (HCMV_clinical_ and HCMV_meta_).

L, LoReTTA; R, Raven; C, Canu; F, Flye; Re, Rebaler.

### 4.2 Effects of genome isoforms

The existence of HCMV and HSV-1 isoforms can result in misassembly errors when two reads originating from different isoforms and traversing *b′a′c′* are joined. In contrast to the other assemblers, LoReTTA and Rebaler consistently produced a single contig for all simulated HCMV and HSV-1 datasets generated from single viral strains. In contrast, the other tools produced fragmented assemblies and rearranged genomes, as confirmed by alignment of the largest contigs to the reference genomes (Supplementary Fig. S1).

The approach taken by LoReTTA, involving reference genome subsampling and local *de novo* assembly of short but highly reliable kmers derived from long reads, played a major role in the accuracy of the assemblies. This is because any variability among long reads due to the occurrence of isoforms is lost when short kmers are selected. In addition, assembling overlapping local contigs in the context of a reference genome steers the genome reconstruction towards the isoform with the standard orientations of U_L_ and U_S_, as also confirmed by the good performance of Rebaler with these datasets.

### 4.3 Effects of datasets derived from simple mixtures

Assembling reads generated from a mixture of two viral strains is challenging because the variation between strains is added to that due to sequencing errors. To test how this influences assembly efficiency, four datasets were simulated by mixing reads generated from HCMV strains Merlin and AD169 in different proportions, with the former in the majority. The two deposited genomes differ by 6,278 substitutions and indels summing to 5,263 nt, with the latter being largely due to a 3,143 nt deletion in the strain AD169 genome. Both LoReTTA and Rebaler reconstructed ≥99 per cent of the strain Merlin genome in a single contig when the proportion of strain AD169 reads was ≤30 per cent (Table 2). Although Flye performed better than LoReTTA for these datasets by producing a smaller number of indels, the assemblies were in general more fragmented, as well as being compromised by artefacts due to the presence of isoforms.

Increasing the proportion of strain AD169 reads to forty per cent resulted in a greater number of substitutions and indels with LoReTTA. This was probably due to coverage depth variation causing strain Merlin to be represented occasionally by a smaller number of reads than strain AD169, resulting in a chimaeric genome reconstruction. Although the lowest accuracy was obtained for this mixed strain dataset, LoReTTA again performed better than the other tools in terms of lower number of mismatches and indels and lower number of misassemblies.

### 4.4 Effects of datasets derived from complex mixtures

Even if processed using a laboratory protocol for increasing the proportion of viral reads, clinical samples with low viral loads may result in datasets in which the majority of reads represent the host genome. In metagenomic datasets, such as those obtained from environmental samples, the situation may be made even more complex by the presence of large numbers of reads from many organisms. Reference-guided *de novo* assembly is likely to be very advantageous in such circumstances. To test this, two series of datasets were simulated by mixing simulated HCMV strain Merlin reads with experimental human reads (to mimic clinical samples enriched in the viral signal) or experimental human microbiome reads (to mimic environmental samples).

For both series, LoReTTA, Rebaler and Raven were capable of assembling the HCMV genome, whereas the other assemblers did not generate an output even after seven days of processing (Table 1). For the clinical series, LoReTTA and Rebaler outperformed Raven in terms of generating a smaller number of nucleotide differences and indels in the reconstructed genomes, and in coping with isoforms (Table 2), with LoReTTA producing a smaller number of indels. Similar results were obtained for the metagenomic series.

### 4.5 Effects of the reference genome

Reference-guided *de novo* assembly requires an adequate level of sequence identity between the reference genome and the reads to be assembled. However, RNA viruses have a high mutation rate, and strains of the same virus may differ significantly in sequence. For example, the genotypes of HCV have been reported to differ by up to thirty-one to thirty-three per cent in genome sequence (Okamoto et al. 1992). This level of variation, together with a high error rate, may greatly reduce the number of reads aligning to a reference genome, which is an early stage in the LoReTTA pipeline (Fig. 2, step B). Although the genomes of DNA virus strains are generally less diverse, poorly conserved regions may occur, for example, in the hypervariable genes of HCMV (Suárez et al. 2019).

Two tests were performed to investigate the effect of a divergent reference genome on the performance of both LoReTTA and Rebaler. First, simulated reads generated from HCV strain H77 (genotype 1) were assembled using the HCV strain HC-J6CH (genotype 2) genome as reference (Supplementary Table S1); the genomes of these strains differ by 31.8 per cent in an MAFFT alignment. Second, simulated reads generated from HCMV strain Merlin were assembled using the HCMV strain Toledo genome (GenBank accession no. GU937742.2) as reference; the two sequences differ by five per cent in an MAFFT alignment, after adjusting for a 14,336 bp inversion in U_L_ in the latter. The minimum overlap percentage was decreased from 70 to 40 and five per cent in the HCMV and HCV experiments, respectively, in order to accommodate the lower level of sequence identity between the reads and the reference genome. In the HCV experiment, LoReTTA generated a single 9,361-nt contig that aligned perfectly to the HCV strain H77 (genotype 1) sequence for ninety-seven per cent of its length, with only the final 285 nucleotides missing (Supplementary Table S3). In the HCMV experiment, LoReTTA generated a single 235,705-nt contig that aligned perfectly to the strain Merlin sequence for 99.7 per cent of its length, although the terminal repeats were erroneously shortened by 686 nt at the 5′ end and extended by 745 nt at the 3′ end. For both datasets, Rebaler produced misassemblies and a higher number of mismatches and indels. These results indicate that LoReTTA retains accuracy even when a divergent reference genome is used.

## 5. Software evaluation on experimental datasets

All the assemblers were tested on experimental PacBio datasets deposited for HBV, HSV-1 and PaP1 (Supplementary Table S2) or generated in this study for HCMV, as described in Section 2.1.2. In general, LoReTTA produced better assemblies in terms of number of contigs, N50 value and number of substitutions and indels (Tables 3 and 4).

**Table 3.** Assembly statistics for four experimental datasets.

Dataset	N50 (nt)					Contigs (no.)					Ambiguous (‘N’) nucleotides (no.)					Assembled nucleotides (no)				
	L	R	C	F	Re	L	R	C	F	Re	L	R	C	F	Re	L	R	C	F	Re
HBV	3,223	—	—	—	2,676	1	—	—	—	1	0	—	—	—	0	3,223	—	—	—	2,676
HSV-1	151,797	28,815	18,382	—	151,254	1	15	69	—	1	994	15	0	—	0	151,754	326,010	930,393	—	151,254
HCMV	236,253	234,751	42,630	199,299	234,164	1	1	36	3	1	0	1	0	0	0	236,253	234,751	463,210	237,725	234,164
PaP1	91,687	91,032	9305	—	91,137	1	1	7	—	1	0	1	0	—	0	91,687	91,032	53,737	—	91,137

–, no assembly was produced when computation was complete (HBV), or assembly had not finished after an impractical length of time (more than seven days), at which point computation was terminated (HSV-1 and PaP1).

L, LoReTTA; R, Raven; C, Canu; F, Flye; Re, Rebaler.

**Table 4.** Comparative statistics for four experimental datasets as determined by the software QUAST using the relevant deposited sequences as references.

Dataset	Genome coverage (%)					Mismatches per 100 kb (no.)					Indels per 100 kb (no.)					Misassemblies (no.)				
	L	R	C	F	Re	L	R	C	F	Re	L	R	C	F	Re	L	R	C	F	Re
HSV-1	98.6	86.2	98.6	—	99.1	0.7	33.5	462.9	—	1.3	10.6	83.7	923.9	—	13.9	0	39	65	—	0
HCMV	100.0	99.3	100.0	99.5	99.1	0.9	0.9	0.9	0.9	1.7	3.4	3.0	7.6	2.6	4.7	0	2	1	0	0
PaP1	99.9	99.3	56.6	—	99.3	1.1	1.1	40.4	—	1.1	1.1	4.4	369.7	—	9.9	0	0	0	—	0

–, no assembly was produced when computation was complete (HBV), or assembly had not finished after an impractical length of time (more than seven days), at which point computation was terminated (HSV-1 and PaP1).

L, LoReTTA; R, Raven; C, Canu; F, Flye; Re, Rebaler.

As for the simulated datasets, only the reference-guided assemblers reconstructed a genome for HBV. LoReTTA produced a 3,223-nt assembly, which entirely covered the genome of its closest relative, HBV strain SH1224-B13 (GenBank accession no. JX661472.1; 3,215 nt), differing by thirty-eight substitutions and featuring one and seven additional nucleotides at the beginning and at the end of the assembled sequence, respectively. According to the relevant metadata (NCBI study PRJNA428411), the reads originated from a mixture of HBV quasispecies, and no deposited genomes were available for comparison.

The HSV-1 PacBio dataset was generated from strain MacIntyre (Jiao et al. 2019). A largely complete sequence for this strain generated from Illumina data had been deposited previously (Szpara et al. 2014; GenBank accession no. KM222720.1; 151,868 nt). Gaps in this sequence were filled subsequently using the PacBio data, thus producing a complete genome (GenBank accession no. MN136523.1; 152,476 nt). The genome (151,797 nt) reconstructed by LoReTTA from the PacBio data differed from the deposited sequence (Table 4 and Supplementary Table S4). Extraneous sequences at the beginning (3 nt) and end (425 nt) of the genome assembled by LoReTTA probably represent shortcomings in the assembly process at the genome termini, as these sequences are well characterized (Davison and Wilkie 1981; Mocarski and Roizman 1981). LoReTTA also failed to reconstruct four repetitive regions, which resulted in the number of ambiguous nucleotides reported in Table 3. In addition, five other differences were observed in the LoReTTA assembly, of which four were deletions (three in homopolymeric tracts) and one was a substitution; each was validated by the greatest number of supporting reads (Supplementary Table S4).

The HCMV PacBio dataset was generated in this study from strain Ig-KG-H2, which was isolated in the presence of HIG and then further passaged in the absence of HIG (Al Qaffas et al. 2020; GenBank accession no. MN274568.2; 236,244 nt), as described in Section 2.1.2. The genome was assembled using LoReTTA and, as the reference, the deposited genome of the virus before passage in the absence of HIG. In comparison with the reference, the reconstructed genome (236,253 nt, Table 3) had two substitutions and a total of nine single nucleotide insertions, one of which was due to an extraneous nucleotide at the end (Supplementary Table S5). Six of the remaining eight insertions represented the same two regions in *a*/*a′* (i.e. there were five different insertions; Table 2). In order to investigate whether these differences had arisen during passage of the virus, rather than from assembly errors, short-read data were generated on an Illumina instrument from the same sample used to generate the long-read data. Variants were validated by counting the number of supporting reads in both the PacBio and Illumina datasets. The PacBio reads confirmed all the variants reported in the LoReTTA assembly, and the Illumina reads validated the two substitutions and two of the five different insertions (Supplementary Table S5). The Illumina data indicated no additional substitutions or indels in the genome reconstructed using LoReTTA, although they did reveal 951- and 2,574-nt deletions in genome subpopulations that were confirmed by the PacBio data. Both of these deletions affected gene RL13, which is known to mutate when virus is passaged in the absence of HIG (Dargan et al. 2010). One of the two substitutions was synonymous and located in gene UL56, and the other was nonsynonymous and located in gene UL130, resulting in the replacement of a C by a W residue at amino acid residue 172. The latter substitution is significant because the UL130 protein is a component of the pentameric complex involved in viral entry into non-fibroblast cells (Wang and Shenk 2005; Hahn et al. 2009). The function of this complex is disadvantageous when virus is passaged in fibroblasts in the absence of HIG, and one of three components (encoded by genes UL128, UL130 and UL131A) invariably mutates under such conditions (Dargan et al. 2010). The crystal structure of the pentameric complex indicates that the substitution in UL130 would remove a disulphide bond normally present between C residues at positions 172 and 207 (Chandramouli et al. 2017). The assembled sequence produced by LoReTTA was submitted to NCBI with the erroneous last nucleotide removed under accession number MT894141.2.

Several misassemblies were observed in the HCMV and HSV-1 sequences generated by Canu and Raven (Table 4), due to the complex genomic rearrangements also observed for simulated datasets (Section 4.2).

The PaP1 PacBio dataset was generated from a strain for which a genome derived from Roche 454 data had been deposited (Lu et al. 2014; GenBank accession no. HQ832595.1; 91,715 nt). Alignment of this sequence with the 91,687-nt genome reconstructed using LoReTTA (Table 3) indicated a few differences (Supplementary Table S6). The absence of 131 nt at the left end and the presence of an extra 82 nt at the right end (corresponding to the 82 nt at the right end of the deposited sequence) may represent shortcomings in the assembly process at the genome termini, which consist of a 1,190 nt direct repeat. A 21 nt insertion and a single substitution elsewhere in the genome were validated by the greatest number of reads.

LoReTTA is limited to extracting reads that map to the reference and therefore, like Rebaler, did not assemble contigs derived from non-target organisms. In contrast, Canu and Raven, which do not rely on a reference, generated an additional twenty-eight and four contigs, respectively, from the HSV-1 dataset (Supplementary Table S7). These were shown by Blastn search not to have originated from HSV-1, but rather from the monkey cell line (Vero) used to grow the virus (Jiao et al. 2019). Similarly, thirty-three of the thirty-six contigs reported by Canu for the HCMV dataset originated from mycoplasma, which is a frequent contaminant of cell cultures; this was also detected in the Illumina reads.

## 6. Conclusion

LoReTTA is a new tool for performing reference-guided *de novo* assembly of long reads generated from viral samples on the PacBio platform. It was designed to deal with the small genomes of viruses, the existence of genome isoforms and the presence of large amounts of nonviral sequences typical of clinical or environmental samples. Our tests on simulated and experimental datasets showed that LoReTTA outperformed three commonly used *de novo* assemblers in terms of the contiguity and accuracy of reconstructed genomes. A similar performance was observed for Rebaler which, however, produced a higher number of misassemblies when a divergent reference genome was used. As is normal in software evaluations, we used these assemblers under default parameters; it is possible that their output could be improved by setting different parameters. Although the software could be used in principle with PacBio data from any organism, the complexity of the protocol is likely to make the assembly step too time-consuming for prokaryotic or eukaryotic genomes. The potential of using multi-threading or grid computing systems to overcome this limitation, and of using long-read data from other platforms (e.g. Oxford Nanopore), will be considered in future releases of LoReTTA.

## Supplementary Material

veab042_Supplementary_Data
