# Supplementary material for: LoReTTA, a user-friendly tool for assembling viral genomes from PacBio sequence data
Source: Virus Evol. 2021 Apr 23;7(1):veab042. doi: 10.1093/ve/veab042 (PMC8111061; doi:10.1093/ve/veab042)
Supplement: veab042_Supplementary_Data [file veab042_supplementary_data.zip › Table S1.docx]

**Table S1**: Data on the viral genomes used in this study.

| **Virus** | **Strain** | **Abbreviation** | **GenBank accession no.** | **Genome size (nt)** | **G+C (%)** |
| --- | --- | --- | --- | --- | --- |
| **Hepatitis B virus** | ayw | HBV_ayw_ | V01460.1 | 3,182 | 48.5 |
| **Hepatitis C virus** | H77 (genotype 1) | HCV_gen1_ | AF009606.1 | 9,646 | 58.2 |
| **Hepatitis C virus** | HC-J6CH (genotype 2) | HCV_gen2_ | AF177036.1 | 9,711 | 56.9 |
| **Severe acute respiratory virus syndrome coronavirus 2** | Wuhan-Hu-1 | SARS-CoV-2_Wuhan_ | MN908947.3 | 29,903 | 38.0 |
| **Pseudomonas aeruginosa phage 1** | -- | PP_PaP1_ | HQ832595.1 | 91,715 | 49.4 |
| **Herpes simplex virus type 1** | KOS | HSV-1_KOS_ | JQ673480.1 | 152,011 | 68.2 |
| **Human cytomegalovirus** | AD169 | HCMV_AD169_ | FJ527563.1 | 231,781 | 57.6 |
| **Human cytomegalovirus** | Merlin | HCMV_Merlin_ | AY446894.2 | 235,646 | 57.5 |

--, not applicable.
