# Supplementary material for: LoReTTA, a user-friendly tool for assembling viral genomes from PacBio sequence data
Source: Virus Evol. 2021 Apr 23;7(1):veab042. doi: 10.1093/ve/veab042 (PMC8111061; doi:10.1093/ve/veab042)
Supplement: veab042_Supplementary_Data [file veab042_supplementary_data.zip › Table S2.docx]

| **Table S2:** Experimental PacBio datasets used in this study. | | | | | |
| --- | --- | --- | --- | --- | --- |
| **Virus** | **SRA accession no.** | **GenBank accession no.** | **Reads (no.)** | **Coverage depth (reads/nt)** | **Average read length (nt)** |
| **HBV** | SRR6438482 | -- | 1,986 | 1,184 | 1,947 |
| **PaP1** | SRR955463, SRR955464 | HQ832595.1 | 17,380 | 194 | 1,037 |
| **HSV-1** | SRR9719185 | MN136523.1 | 311,199 | 4,810 | 3,228 |
| **HCMV** | ERR5052619 | -- | 29,340 | 188 | 1,627 |
| --, not applicable. | | | | | |
