# Supplementary material for: LoReTTA, a user-friendly tool for assembling viral genomes from PacBio sequence data
Source: Virus Evol. 2021 Apr 23;7(1):veab042. doi: 10.1093/ve/veab042 (PMC8111061; doi:10.1093/ve/veab042)
Supplement: veab042_Supplementary_Data [file veab042_supplementary_data.zip › Table S3.docx]

| **Dataset** | **Assembler** | **Reference** | **N50** | **Contigs (no.)** | **Ambiguous (‘N’) nucleotides (no.)** | **Assembled nucleotides (no.)** | **Genome coverage (%)** | **Mismatches per 100 kb (no.)** | **Indels per 100 kb (no.)** | **Misassemblies (no.)** |
| --- | --- | --- | --- | --- | --- | --- | --- | --- | --- | --- |
| **HCV_gen1_^100^** | LoReTTA | HCV_gen2_ | 9,361 | 1 | 0 | 9,361 | 97 | 0 | 0 | 0 |
| **HCV_gen1_^100^** | Rebaler | HCV_gen2_ | 6,968 | 1 | 0 | 6,968 | 72.2 | 488.2 | 186.7 | 1 |
| **HCMV_Merlin_^100^** | LoReTTA | HCMV_Toledo_ | 234,564 | 1 | 0 | 234,564 | 99.5 | 0 | 0 | 0 |
| **HCMV_Merlin_^100^** | Rebaler | HCMV_Toledo_ | 233,780 | 1 | 0 | 233,780 | 99.2 | 0 | 1.7 | 2 |

**Table S3:** Assembly statistics for two simulated datasets assembled using a divergent genome as reference (also used to compute the comparative statistics with QUAST)
