# Supplementary material for: LoReTTA, a user-friendly tool for assembling viral genomes from PacBio sequence data
Source: Virus Evol. 2021 Apr 23;7(1):veab042. doi: 10.1093/ve/veab042 (PMC8111061; doi:10.1093/ve/veab042)
Supplement: veab042_Supplementary_Data [file veab042_supplementary_data.zip › Table S4.docx]

| **Type** | **Position (nt)^a^** | **Difference** | | **Supporting PacBio reads (no.)** | |
| --- | --- | --- | --- | --- | --- |
|  |  | **LoReTTA** | **Deposited** | **LoReTTA** | **Deposited** |
| **Deletion (G-tract)** | 6,220 | - | G | 2,700 | 1,063 |
| **Deletion (C-tract)** | 6,602 | - | C | 2,193 | 1,584 |
| **Deletion (A-tract)** | 7,106 | - | A | 2,657 | 1,060 |
| **Substitution** | 16,730 | A | G | 2,694 | 1,585 |
| **Unassembled repetitive region** | 117,315-117,648 | -- | -- | -- | -- |
| **Unassembled repetitive region** | 126,371-127,379 | -- | -- | -- | -- |
| **Deletion** | 141,504 | - | C | 1,860 | 10 |
| **Unassembled repetitive region** | 151,246-151,804 | -- | -- | -- | -- |
| **Unassembled repetitive region** | 152,203-152,360 | -- | -- | -- | -- |
| ^a^Relative to an alignment between the LoReTTA and deposited genomes made using MAFFT under default parameters. | | | | | |
| -, deleted; --, not applicable. |  |  |  |  |  |

**Table S4:** Numbers of reads supporting the differences between the HSV-1 genome reconstructed using LoReTTA and the deposited genome (GenBank accession no. MN136523.1).
