# Supplementary material for: LoReTTA, a user-friendly tool for assembling viral genomes from PacBio sequence data
Source: Virus Evol. 2021 Apr 23;7(1):veab042. doi: 10.1093/ve/veab042 (PMC8111061; doi:10.1093/ve/veab042)
Supplement: veab042_Supplementary_Data [file veab042_supplementary_data.zip › Table S5.docx]

| **Type** | **Position (nt)^a^** | **Difference** | | **Supporting PacBio reads (no.)** | | **Supporting Illumina reads (no.)** | |
| --- | --- | --- | --- | --- | --- | --- | --- |
|  |  | **LoReTTA** | **Deposited** | **LoReTTA** | **Deposited** | **LoReTTA** | **Deposited** |
| **Insertion (G-tract)^b^** | 300 | G | - | 358 | 90 | 1296 | 1020 |
| **Insertion (G-tract)^c^** | 441 | G | - | 291 | 244 | 160 | 1278 |
| **Substitution** | 86,552 | C | G | 151 | 5 | 2547 | 141 |
| **Substitution** | 177,124 | C | A | 177 | 0 | 2205 | 4 |
| **Insertion (G-tract)** | 231,718 | G | - | 50 | 7 | 346 | 318 |
| **Insertion (G-tract)** | 231,961 | G | - | 26 | 20 | 200 | 262 |
| **Insertion** | 236,253 | G | - | -- | -- | -- | -- |
| ^a^Relative to an alignment between the LoReTTA and deposited genomes made using MAFFT under default parameters. | | | | | | | |
| ^b^Insertion in *a* that is also present in the other copies of this sequence (positions 195,349 and 235,846). | | | | | | | |
| ^c^Insertion in *a* that is also present in the other copies of this sequence (positions 195,208 and 235,990). | | | | | | | |
| -, deleted; --, not applicable due to an additional nt at the end of the assembly for which supporting reads could not be enumerated by the method used. | | | | | | | |

**Table S5:** Numbers of reads supporting the differences between the HCMV genome reconstructed using LoReTTA and the deposited genome**.**
