# Supplementary material for: LoReTTA, a user-friendly tool for assembling viral genomes from PacBio sequence data
Source: Virus Evol. 2021 Apr 23;7(1):veab042. doi: 10.1093/ve/veab042 (PMC8111061; doi:10.1093/ve/veab042)
Supplement: veab042_Supplementary_Data [file veab042_supplementary_data.zip › Table S6.docx]

**Table S6:** Number of reads supporting the differences between the PaP1 genome reconstructed using LoReTTA and the deposited genome**.**

| **Type** | **Position (nt)^a^** | **Difference** | | **Supporting PacBio reads (no.)** | |
| --- | --- | --- | --- | --- | --- |
|  |  | **LoReTTA** | **Deposited** | **LoReTTA** | **Deposited** |
| **Insertion** | 42,208 | CGGTGCTCCATGGTACTCGGT | - | 80 | 0 |
| **Substitution** | 32,456 | C | A | 92 | 7 |
| ^a^Relative to an alignment between the LoReTTA and deposited genomes made using MAFFT under default parameters. | | | | | |
| -, deleted. | | | | | |
