# Supplementary material for: LoReTTA, a user-friendly tool for assembling viral genomes from PacBio sequence data
Source: Virus Evol. 2021 Apr 23;7(1):veab042. doi: 10.1093/ve/veab042 (PMC8111061; doi:10.1093/ve/veab042)
Supplement: veab042_Supplementary_Data [file veab042_supplementary_data.zip › Table S7.docx]

**Table S7** Number of assembled contigs sharing no similarity with the targeted viral sequence.

| **Dataset** | **Contigs (no.)** | | | | |
| --- | --- | --- | --- | --- | --- |
|  | **LoReTTA** | **Raven** | **Canu** | **Flye** | **Rebaler** |
| **HBV** | 0 | -- | -- | -- | 0 |
| **PaP1** | 0 | 0 | 0 | -- | 0 |
| **HSV-1** | 0 | 4 | 28 | -- | 0 |
| **HCMV** | 0 | 0 | 33 | 0 | 0 |

--, no assembly was generated.
