# Supplementary figures and images for: LoReTTA, a user-friendly tool for assembling viral genomes from PacBio sequence data
Source: Virus Evol. 2021 Apr 23;7(1):veab042. doi: 10.1093/ve/veab042 (PMC8111061; doi:10.1093/ve/veab042)

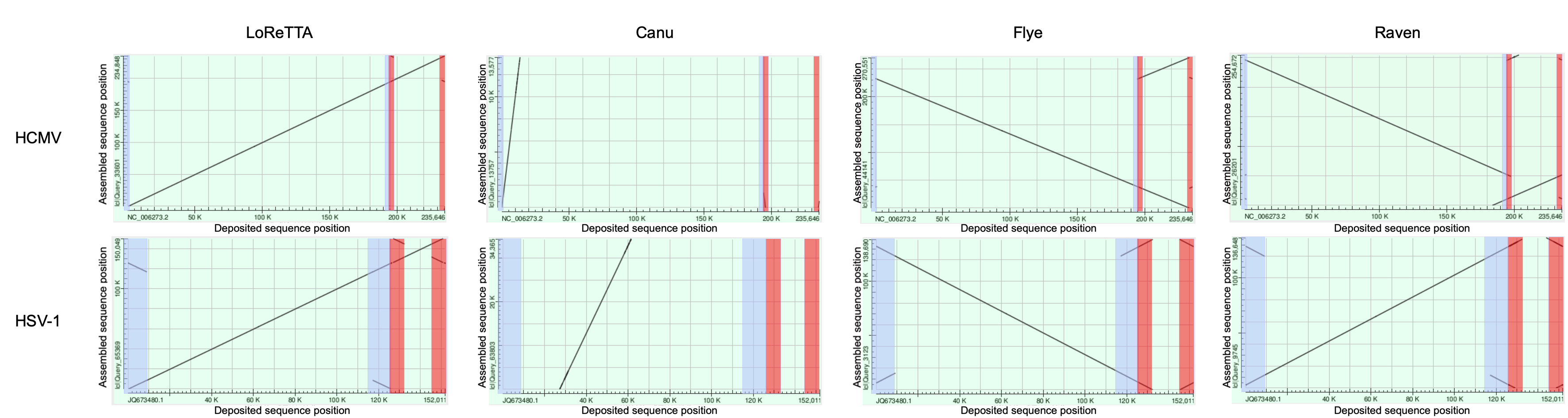

Supplement: veab042_Supplementary_Data [file veab042_supplementary_data.zip › Figure S1.jpg]
